# Supplementary material for: Circular RNA profiling in the oocyte and cumulus cells reveals that circARMC4 is essential for porcine oocyte maturation
Source: Aging (Albany NY). 2019 Sep 28;11(18):8015–34. doi: 10.18632/aging.102315 (PMC6781969; doi:10.18632/aging.102315)
Supplement: Supplementary Figures [file aging-11-102315-s006.pdf]

SUPPLEMENTARY FIGURES

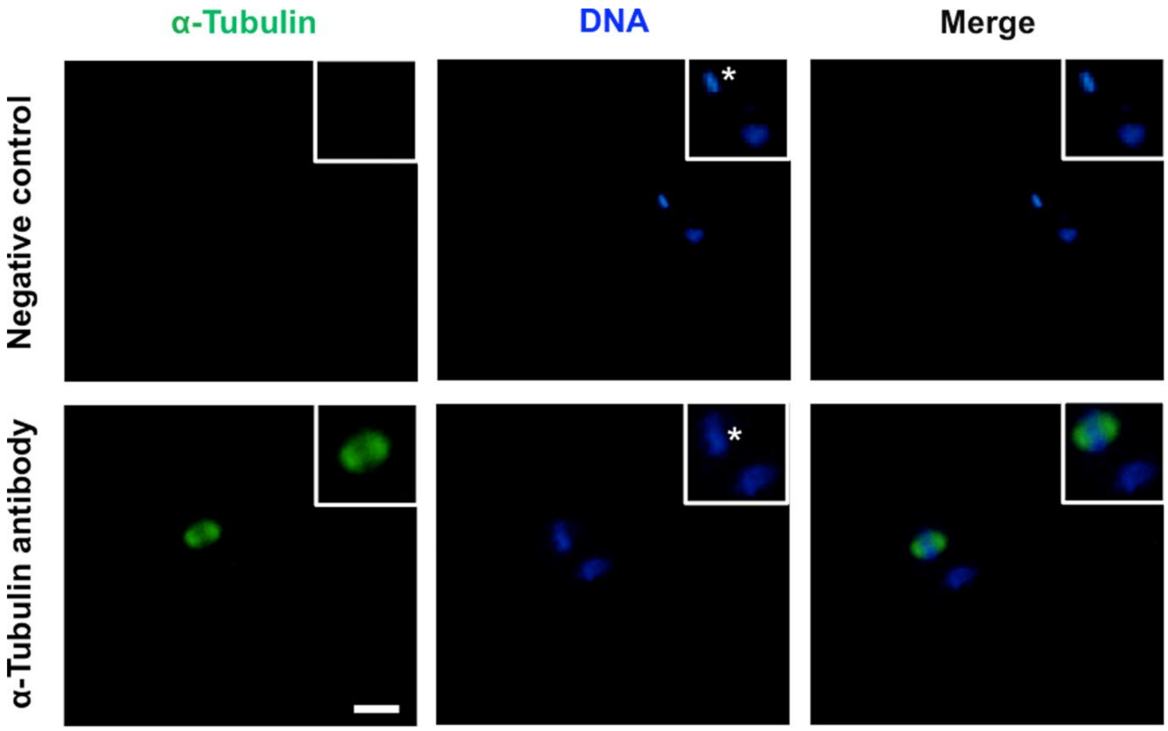

Supplementary Figure 1. Validation of the specificity of α-tubulin antibody. MII oocytes were incubated with the diluent α-tubulin antibody in the immunofluorescence staining protocol while MII oocytes incubated with the mouse serum (Yeasen, 36118ES03) replacing α-tubulin antibody serve as a negative control. DNA was stained with DAPI (blue). Shown are representative images obtained using confocal laser-scanning microscopy. Right panel in each group shows the merged images between α-TUBULIN and DNA. White square insets indicate both spindles and chromosomes at high magnification. Asterisks mark chromosomes. Scale bar: 50 µm.

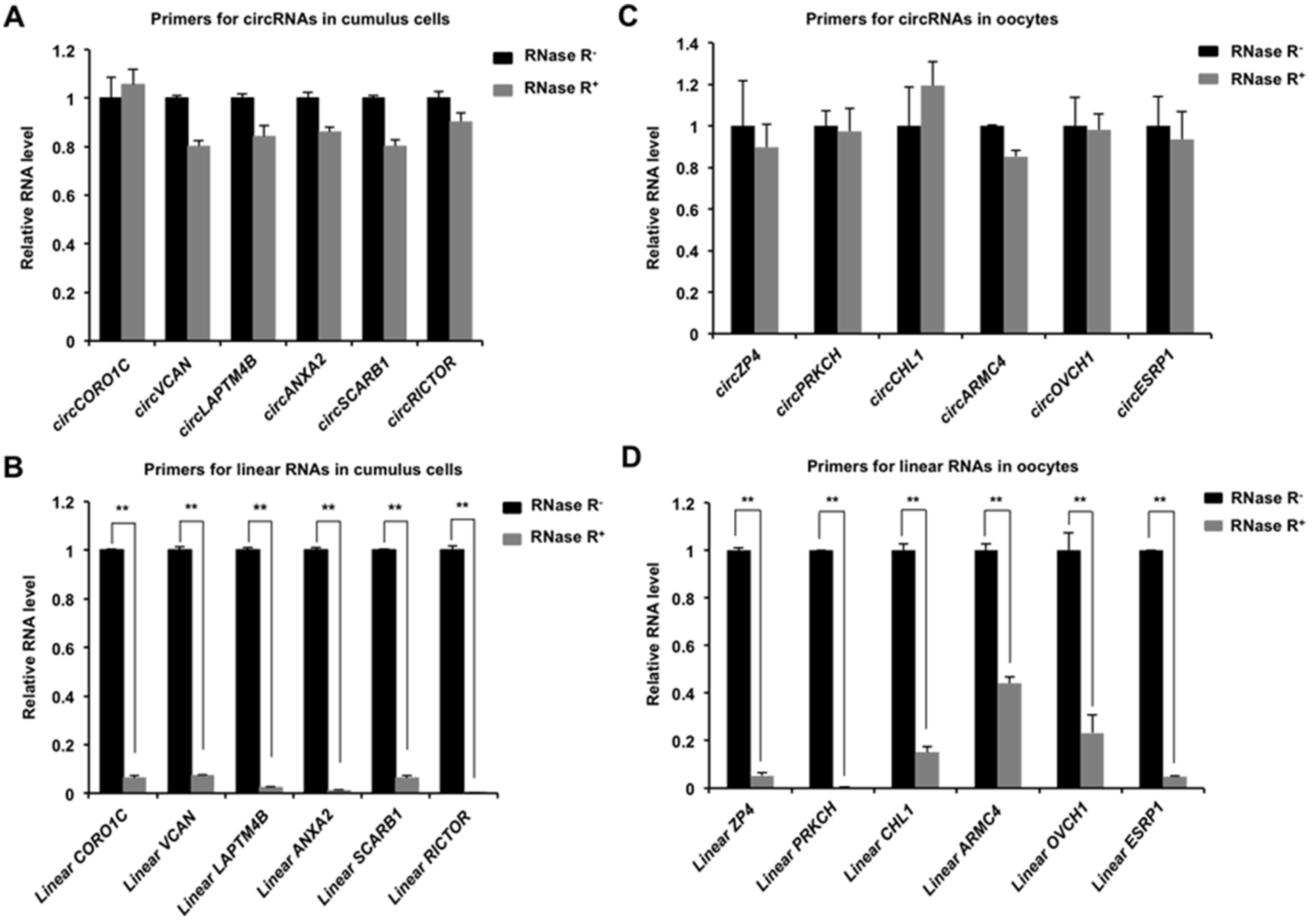

Supplementary Figure 2. Expression of circRNAs and their corresponding linear RNAs in MCC and GV oocytes treated with or without RNase R. The several selected circRNAs and their corresponding linear RNAs were chosen from top differentially expressed circRNAs in both cumulus cells and oocytes. Total RNAs were extracted from MCC and GV oocytes, and then were treated with RNase R. The total RNAs without RNase R treatment serve as a control. Relative expression levels of the indicated circRNAs (**A**, **C**) and the corresponding linear RNAs (**B**, **D**) before and after RNase R treatment were determined by qPCR. The data were normalized against endogenous housekeeping gene *EF1α1,* and the value for both the MCC and the GV oocyte in the control group was set as one. The data are shown as mean ± S.E.M. Statistical analysis was performed using *t*-student test. Values with asterisks vary significantly, ***P* < 0.01.

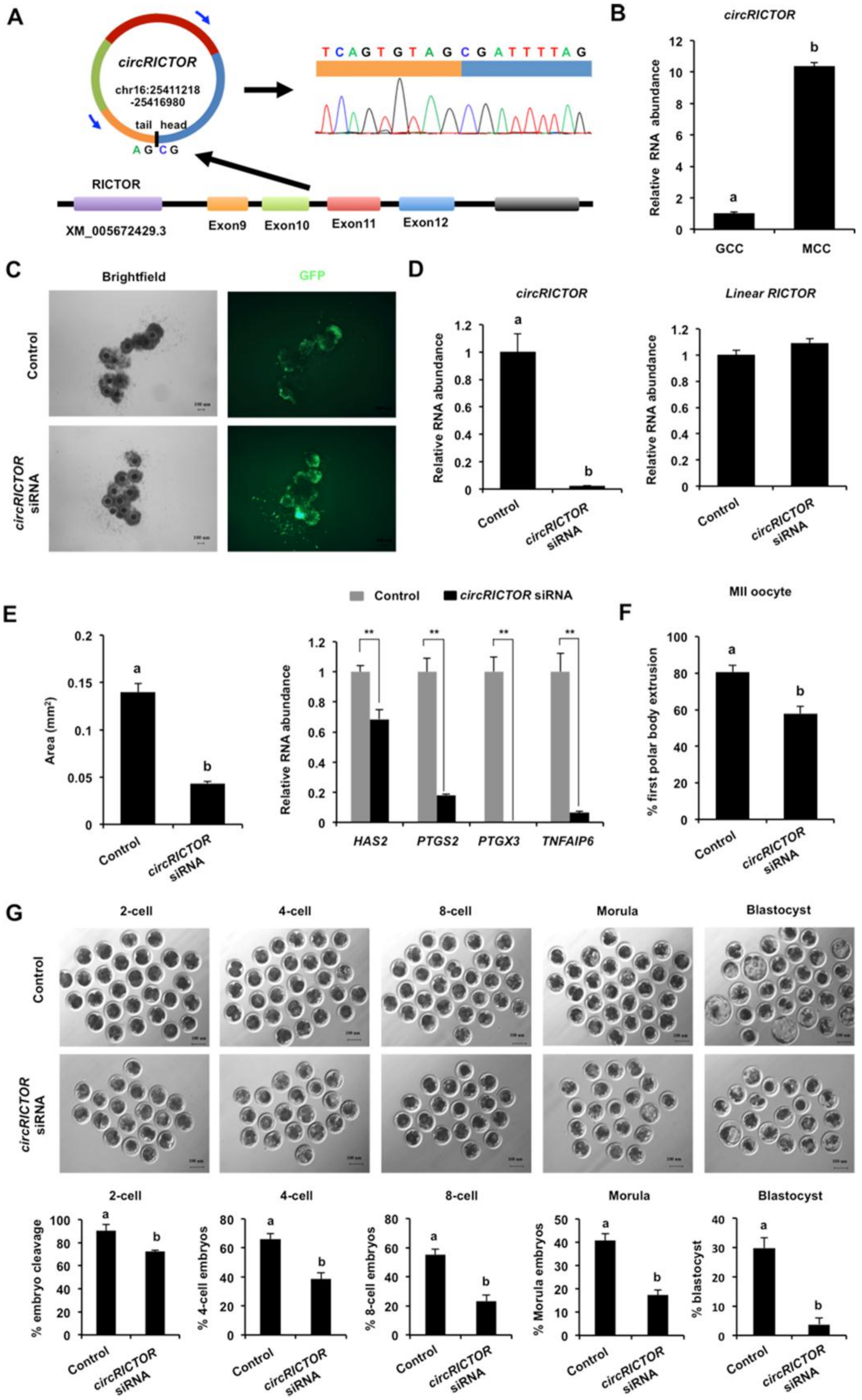

Supplementary Figure 3. Effect of *circRICTOR* knockdown in cumulus cells on oocyte meiotic maturation and early embryo development. (**A**) Schematic illustration showed the RICTOR exon 9, 10, 11 and 15 circularization forming *circRICTOR* (blue arrow). The presence of *circRICTOR* was validated by qPCR, followed by Sanger sequencing. Black arrow represents "head-to-tail" *circRICTOR* splicing sites. (**B**) *CircRICTOR* expression in cumulus cells before and after oocyte maturation. (**C**) Representative images of cumulus-oocyte complexes transduced by lentivirus. (**D**) Expression levels of *circRICTOR* and linear *RICTOR* in cumulus cells after oocyte maturation. (**E**) Effect of *circRICTOR* knockdown on the expression of genes associated with cumulus expansion. The data are shown as mean ± S.E.M. Statistical analysis was performed using *t*-student test. Values with asterisks or different letters vary significantly, $**P < 0.01$. (**F**) *circRICTOR* knockdown reduces the rate of oocyte meiotic maturation. (**G**) *circRICTOR* knockdown impairs early embryo development.
