# Supplementary material for: Circular RNA profiling in the oocyte and cumulus cells reveals that circARMC4 is essential for porcine oocyte maturation
Source: Aging (Albany NY). 2019 Sep 28;11(18):8015–34. doi: 10.18632/aging.102315 (PMC6781969; doi:10.18632/aging.102315)
Supplement: Supplementary Table 1 [file aging-11-102315-s002.docx]

**Supplementary Table 1. Porcine-specific primer and siRNA sequences used in this study.**

**Primers of circRNAs in cumulus cell**

| **Gene** | **Primer sequences (5'－3')** | **Product size** | | **GenBank**  **accession no.** |
| --- | --- | --- | --- | --- |
| *circCORO1C* | F: ACAAGTCCTACCCTACAGTATG | 94 bp | XM_005670752.3 | |
|  | R: TCTGAACCGCTGGCAATG |  |  |  |
| *circVCAN* | F: CTCCGTGTCATTGGCTGTG | 114 bp | NM_001206429.1 | |
|  | R: GGTGGTAAGGTAGGCATAGTTG |  |  |  |
| *circAPTM4B* | F: GCTACCGATACATCAATGG | 160 bp | XM_005655356.3 | |
|  | R: AAGTCACCTCCTAGTTCAG |  |  |  |
| *circANXA2* | F: AGCACTCCACACCCGCAAG | 88 bp | NM_001005726.1 | |
|  | R: GCCGTCTCAATGTTCAGAGCATC |  |  |  |
| *circSCARB1* | F: GCTCAACAACTCCGATTC | 125 bp | NM_213967.1 | |
|  | R: GCCTATCCTCCATCATCAC |  |  |  |
| *circ RICTOR* | F: TCCGTCTTCCTCTACCTGTTG | 144 bp | XM_005672429.3 | |
|  | R: CCTTGCTTCTCTGTCTTCTTTAAG |  |  |  |

**Primers of circRNAs in oocyte**

| **Gene** | **Primer sequences (5'－3')** | **Product size** | | **GenBank**  **accession no.** |
| --- | --- | --- | --- | --- |
| *circZP4* | F: CTATCCGTCACCGAACAG | 135 bp | NM_214045.1 | |
|  | R: CCGAAGCAATTTCACCAC |  |  |  |
| *circPRKCH* | F: CCTTTCCTCACCCAGTTG | 158 bp | XM_021062478.1 | |
|  | R: AGTCCTTGTCGCATTATCC |  |  |  |
| *circCHL1* | F: CTGTTCAGTGGAAGAAGAGTAATC | 169 bp | XM_021069242.1 | |
|  | R: GAAGGAGGCAGCCCAGAAAGAATG |  |  |  |
| *circARMC4* | F: GCATCACCATGCACGAGAAC | 132 bp | XM_021064726.1 | |
|  | R: CAGCACACACACTTGCCAAA |  |  |  |
| *circOVCH1* | F: TGGCATTTGAAGACTTTAGTATTG | 189 bp | XM_021092126.1 | |
|  | R: CCAGGGAAGCAAGGTTCG |  |  |  |
| *circESRP1* | F: TGAACTACACAGCATATTATC | 187bp | XM_021089092.1 | |
|  | R: CAGAAAGTCCAGGATGTC |  |  |  |

**Primers of linear mRNAs in cumulus cell**

| **Gene** | **Primer sequences (5'－3')** | **Product size** | | **GenBank accession no.** |
| --- | --- | --- | --- | --- |
| *Linear CORO1C* | F: TTGTCGCCATAATCATAGAAGC | 101 bp | XM_005670752.3 | |
|  | R: GCCACATACTGTAGGGTAGG |  |  |  |
| *Linear VCAN* | F: CCTTACCACCCAGTTACAACAC | 166 bp | NM_001206429.1 | |
|  | R: GTAGGCACCGACACTCTTCC |  |  |  |
| *Linear LAPTM4B* | F: GTGCTGTTGATTCTGCTGAG | 181bp | XM_005655356.3 | |
|  | R: CTGCGTGTTGCTTGTATGC |  |  |  |
| *Linear ANXA2* | F: GAGGTCACCATCGTCAAC | 132 bp | NM_001005726.1 | |
|  | R: CGGAAGCGTCATACTGAG |  |  |  |
| *Linear SCARB1* | F: CCCTCATACATCTCATCAATAAG | 112 bp | NM_213967.1 | |
|  | R: GTGAACACGGTGAAGAGC |  |  |  |
| *Linear RICTOR* | F: AAATATGGAAATAAGGCGAGGTC | 80 bp | XM_005672429.3 | |
|  | R: ACTCTTCAGTCACAACAGGTAG |  |  |  |
| *Linear HAS2* | F: GAAGTCATGGGCAGGGACAATTC | 407bp | NM_214053 | |
|  | R: TGGCAGGCCCTTTCTATGTTA |  |  |  |
| *Linear PTGS2* | F: ATGATCTACCCGCCTCACAC | 279bp | AF_207824 | |
|  | R: GCAGCTCTGGGTCAAACTTC |  |  |  |
| *Linear PTGX3* | F: TCAGTGCCTGCATTTGGGTC | 225bp | GQ_412351 | |
|  | R: CTACATGCCCTTGTTCAGAA |  |  |  |
| *Linear TNFAIP6* | F: TCATAACTCCATATGGCTTGAAC | 396bp | NM_001159607.1 | |
|  | R: TCTTCGTACTCATTTGGGAAGCC |  |  |  |
| *Linear EF1α1* | F: ATTGTTGCTGCTGGTGTTG | 161 bp | NM_001097418.2 | |
|  | R: TCATATCTCTTCTGGCTGTAGG |  |  |  |

**Primers of linear mRNAs in oocyte**

| **Gene** | **Primer sequences (5'－3')** | **Product size** | | **GenBank accession no.** |
| --- | --- | --- | --- | --- |
| *Linear ZP4* | F: ACGCTATGGCTCCTACAATG | 95 bp | NM_214045.1 | |
|  | R: CTGTTCGGTGACGGATAGAGAC |  |  |  |
| *Linear PRKCH* | F: AACACTGGCTTAGAGTCTTC | 195 bp | XM_021062478.1 | |
|  | R: TCCTTCTTTGGTGCTTTCC |  |  |  |
| *Linear CHL1* | F: CCAAAAGTAAGAGGAGACATTAAG | 89 bp | XM_021069242.1 | |
|  | R: CTTCTTCCACTGAACAGCATC |  |  |  |
| *Linear ARMC4* | F: GAAATGGTTCGTTCCTTTGTTG | 102 bp | XM_021064726.1 | |
|  | R: ATTGGTAATAGCAGCACACAC |  |  |  |
| *Linear OVCH1* | F: ATGCTCAAGAGTACGAACC | 168 bp | XM_021092126.1 | |
|  | R: TTCAATACTAAAGTCTTCAAATGC |  |  |  |
| *Linear ESRP1* | F: GCTGAGGAGATGAACTTTGTG | 129 bp | XM_021089092.1 | |
|  | R: AGGAATAACTGCTGCTGGAG |  |  |  |
| *Linear EF1α1* | F: ATTGTTGCTGCTGGTGTTG | 161 bp | NM_001097418.2 | |
|  | R: TCATATCTCTTCTGGCTGTAGG |  |  |  |

**Information of *circARMC4* siRNA sequence**

| ***circARMC4* siRNA** | **Sense (5'－3')** | **Antisense (5'－3')** |
| --- | --- | --- |
| *circARMC4 siRNA 1* | CGGCGCUGUGAAGGAUGCUTT | AGCAUCCUUCACAGCGCCGTT |
| *circARMC4 siRNA 2* | GCUGUGAAGGAUGCUGGAGTT | CUCCAGCAUCCUUCACAGCTT |
| *circARMC4 siRNA 3* | GUGAAGGAUGCUGGAGAAATT | UUUCUCCAGCAUCCUUCACTT |

**Information of *circRICTOR* recombinant lentivirus sequences**

| ***CircRICTOR*** | **Sense (5'－3')** | **Antisense (5'－3')** |
| --- | --- | --- |
| *circRICTOR* | ACTACTCAGTGTAGCGAATTT | TTCTCCGAACGTGTCACGT |
