# Supplementary material for: Circular RNA profiling in the oocyte and cumulus cells reveals that circARMC4 is essential for porcine oocyte maturation
Source: Aging (Albany NY). 2019 Sep 28;11(18):8015–34. doi: 10.18632/aging.102315 (PMC6781969; doi:10.18632/aging.102315)
Supplement: Supplementary Table 2 [file aging-11-102315-s003.pdf]

Supplementary Table 2. Summary of sequencing results in porcine cumulus cells and oocytes at two maturational stages.

Sample name	Raw reads	Valid reads	Clean bases(G)	Clean Ratio(reads)	Q20%	Q30%	GC content%
Pre-mature COC1	55690890	54111584	8.12G	97.16	98.88	90.77	60
Pre-mature COC2	80463120	78580046	11.79G	97.66	99.04	92.89	61
Pre-mature COC3	62790698	61122920	9.17G	97.34	99.13	91.86	60
GCC1	91383614	87290862	13.09G	95.52	98.85	93.72	62
GCC2	90896402	86742610	13.01G	95.43	98.78	93.93	62
GCC3	80986920	78284342	11.74G	96.66	98.86	94.23	61
Mature COC1	60185534	58543068	8.78G	97.27	98.89	91.25	62
Mature COC2	59305888	57515622	8.63G	96.98	98.95	91.35	61
Mature COC3	58306736	56390364	8.46G	96.71	98.76	90.35	61
MCC1	59697644	57701474	8.66G	96.66	98.37	88.99	62
MCC2	63826660	62080180	9.31G	97.26	98.74	90.01	61
MCC3	67342322	65078844	9.76G	96.64	99.1	91.22	61.5
Total	830876428	803441916					
